# Knockdown of Human *TCF4* Affects Multiple Signaling Pathways Involved in Cell Survival, Epithelial to Mesenchymal Transition and Neuronal Differentiation

**DOI:** 10.1371/journal.pone.0073169

**Published:** 2013-08-23

**Authors:** Marc P. Forrest, Adrian J. Waite, Enca Martin-Rendon, Derek J. Blake

**Affiliations:** 1 Institute of Psychological Medicine and Clinical Neurosciences, MRC Centre for Neuropsychiatric Genetics and Genomics, School of Medicine, Cardiff University, Cardiff, United Kingdom; 2 Nuffield Division of Clinical Laboratory Sciences, Radcliffe Department of Medicine, University of Oxford, Oxford, United Kingdom; 3 Stem Cell Research Laboratory, NHS Blood and Transplant, John Radcliffe Hospital, Oxford, United Kingdom; CNRS UMR7275, France

## Abstract

Haploinsufficiency of *TCF4* causes Pitt-Hopkins syndrome (PTHS): a severe form of mental retardation with phenotypic similarities to Angelman, Mowat-Wilson and Rett syndromes. Genome-wide association studies have also found that common variants in *TCF4* are associated with an increased risk of schizophrenia. Although TCF4 is transcription factor, little is known about TCF4-regulated processes in the brain. In this study we used genome-wide expression profiling to determine the effects of acute TCF4 knockdown on gene expression in SH-SY5Y neuroblastoma cells. We identified 1204 gene expression changes (494 upregulated, 710 downregulated) in TCF4 knockdown cells. Pathway and enrichment analysis on the differentially expressed genes in TCF4-knockdown cells identified an over-representation of genes involved in TGF-β signaling, epithelial to mesenchymal transition (EMT) and apoptosis. Among the most significantly differentially expressed genes were the EMT regulators, *SNAI2* and *DEC1* and the proneural genes, *NEUROG2* and *ASCL1*. Altered expression of several mental retardation genes such as *UBE3A* (Angelman Syndrome), *ZEB2* (Mowat-Wilson Syndrome) and *MEF2C* was also found in TCF4-depleted cells. These data suggest that TCF4 regulates a number of convergent signaling pathways involved in cell differentiation and survival in addition to a subset of clinically important mental retardation genes.

## Introduction

Transcription factor 4 (TCF4) is a basic helix-loop-helix (bHLH) transcription factor involved in neurodevelopment and dendritic cell diversification in the immune system [1–5]. Mounting genetic and biological evidence supports a crucial role for TCF4 in healthy brain function. Mutations in *TCF4* cause Pitt-Hopkins syndrome (PTHS); a severe mental retardation syndrome associated with a facial gestalt, breathing abnormalities, visual problems, delayed speech development and seizures [6–8]. PTHS is caused by deletions, nonsense and missense mutations in the *TCF4* gene on human chromosome 18, resulting in haploinsufficiency [6–9]. The majority of *TCF4* missense mutations cluster in the bHLH domain of the protein where they attenuate transcription and can impair protein–protein interactions [6,8,10–12]. In addition to PTHS, balanced chromosomal abnormalities disrupting *TCF4* and copy number variants have been found in patients with autism and neurodevelopmental disorders that were previously undiagnosed with PTHS [13].

In addition to rare mutations, common variants (single nucleotide polymorphisms, SNP) in *TCF4* are associated with an increased risk of schizophrenia [14,15]. Genome-wide association studies found that a SNP (rs9960767) located in intron 3 of *TCF4* was associated with schizophrenia (*P* = 4.1 x 10^-9^), surpassing the statistical threshold for genome-wide significance [14,15]. Additional *TCF4* variants have also been associated with schizophrenia providing further support for *TCF4* as a schizophrenia risk gene [1]. Interestingly, the rs9960767 risk allele is correlated with impaired sensorimotor gating and cognitive performance, mirroring established schizophrenia endophenotypes [16,17]. Recently, Wirgenes and colleagues found that psychosis was associated with elevated *TCF4* transcript levels and that *TCF4* risk variants were associated with a range of clinical, cognitive and brain morphological abnormalities [18]. Multiple alternatively spliced TCF4 isoforms have been described, however the role of the different spliced forms on transcription has not been extensively studied [19,20]. It is therefore possible that SNPs associated with schizophrenia may differentially regulate the expression of one or more TCF4 isoforms leading to subtle changes in critical neurodevelopmental pathways.

TCF4, TCF12 (HEB), TCF3 (E2A) form the class I bHLH transcription factors in humans [21]. TCF4 interacts with a potentially large repertoire of transcription factors including the products of proneural genes such as ASCL1, ATOH1 and NEUROD1 to regulate neurogenesis and cell type specification in the developing brain [5,15,22,23]. ASCL1, ATOH1 and NEUROD1 are bHLH transcription factors that form obligatory heterodimers with TCF4 (and other E-proteins) to regulate gene expression at E-box containing promoters. Heterodimers bind directly to E-boxes that have the consensus sequence “CANNTG” allowing them to regulate gene expression. Additionally, the structurally related ID (inhibitors of differentiation) proteins such as ID2, are dominant-negative regulators of TCF4. ID proteins form inactive heterodimers with TCF4 preventing the sequestered protein from binding E-boxes or interacting with other transcriptional activators thereby maintaining cells in an undifferentiated state. During neurodevelopment, the balance of E-proteins, proneural and ID proteins in a cell are therefore an important regulatory step in determining cell fate [24].

The role of TCF4 in brain development and behavior has also been studied in mouse models. Homozygous *Tcf4* knockout mice do not survive past birth and although gross brain structure and size does not seem to be affected, there are specific regions of the pontine nucleus where cells fail to migrate to their correct positions [5]. Furthermore, adult mice moderately over-expressing TCF4 in the brain have behavioural abnormalities including deficits in sensory-motor gating and cognitive performance, reminiscent of certain schizophrenia endophenotypes [22].

Although TCF4 has been studied in the context of neurodevelopment and behavior, very little is known about TCF4 regulated genes and processes in the developing and adult nervous system. In this study we use small interfering RNA (siRNA) to acutely knockdown *TCF4* in the SH-SY5Y neuronal cell line. Using microarrays and conservative pathway analysis we find that TCF4 knockdown is associated with robust gene expression changes in multiple, convergent signaling pathways.

## Results

### Knockdown of TCF4 in SH-SY5Y cells

**Figure 1:**
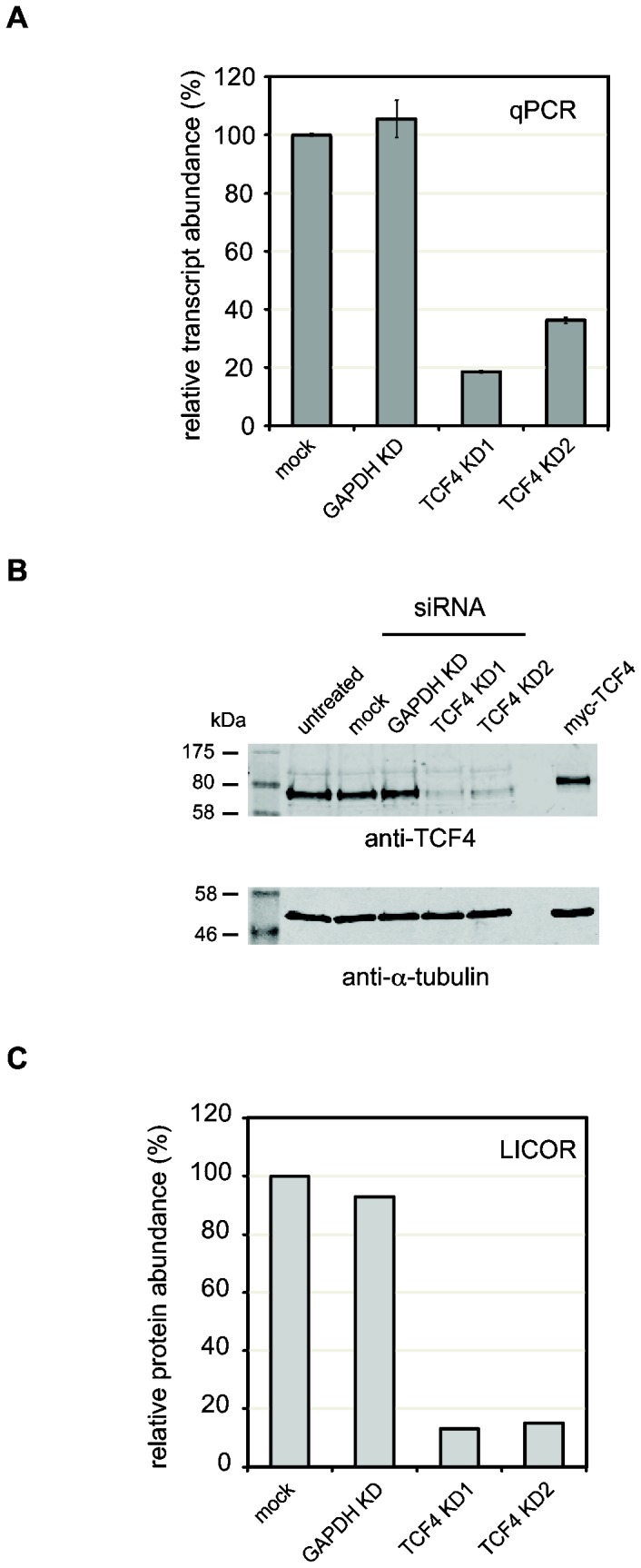
siRNA-mediated knockdown of *TCF4* in SH-SY5Y cells. SH-SY5Y cells were transfected with siRNA oligonucleotides targeting TCF4 (KD1 and KD2) or GAPDH. After 72h, RNA and protein were extracted to assess the knockdown efficiency. (A) Primers complimentary to a constitutive exon present in all TCF4 isoforms (exon 13) were used to measure overall transcript abundance by qPCR in three biological replicates. *TCF4* knockdown efficiency was similar with both KD1 and KD2 whereas the control GAPDH KD did not affect *TCF4* transcript levels. (B) Western blots of protein lysates prepared from siRNA-treated SH-SY5Y cells demonstrated that KD1 and KD2 reduced TCF4 levels whilst GAPDH knockdown had no apparent effect. α-tubulin was used as a loading control and for normalization. (C) LI-COR quantitation of TCF4 protein levels in siRNA-treated cells. The levels of TCF4 in each experiment were quantified and normalized to α-tubulin as described previously [54,55]. In agreement with the qPCR results, siRNA treatment reduced TCF4 levels to approximately 20% of wild-type compared to mock or GAPDH KD-treated cells.

To determine the effects of *TCF4* depletion on gene expression in a cell line of neuronal origin, we tested a series of siRNA duplexes for their efficacy in knocking down *TCF4* in SH-SY5Y cells. SH-SY5Y cells were chosen because they express high levels of TCF4 and can be efficiently transfected with single siRNA duplexes to achieve a robust knockdown. Quantitative PCR (qPCR) and quantitative western blots were used to test the efficacy of each duplex to knockdown *TCF4*. qPCR analysis of each knockdown showed that KD1 (exon 12) caused an 80% reduction of the TCF4 transcript whereas KD2 (exon 19) reduced *TCF4* expression by 62% compared to mock transfected cells (Figure 1A). Importantly, siRNAs targeting *GAPDH* (exons 1 and 2) and mock transfection had no significant effect on the level of the *TCF4* transcript (Figure 1A). Reduced levels of the TCF4 protein was also verified by quantitative western blotting (Figure 1B and 1C). Knockdown of *TCF4* with TCF4 KD1 and KD2 resulted in a greater than 80% reduction in proteins levels with both duplexes. KD2 was less effective than KD1 at knocking down the TCF4 protein reflecting the qPCR data.

### Microarray analysis of TCF4 knockdown

**Figure 2:**
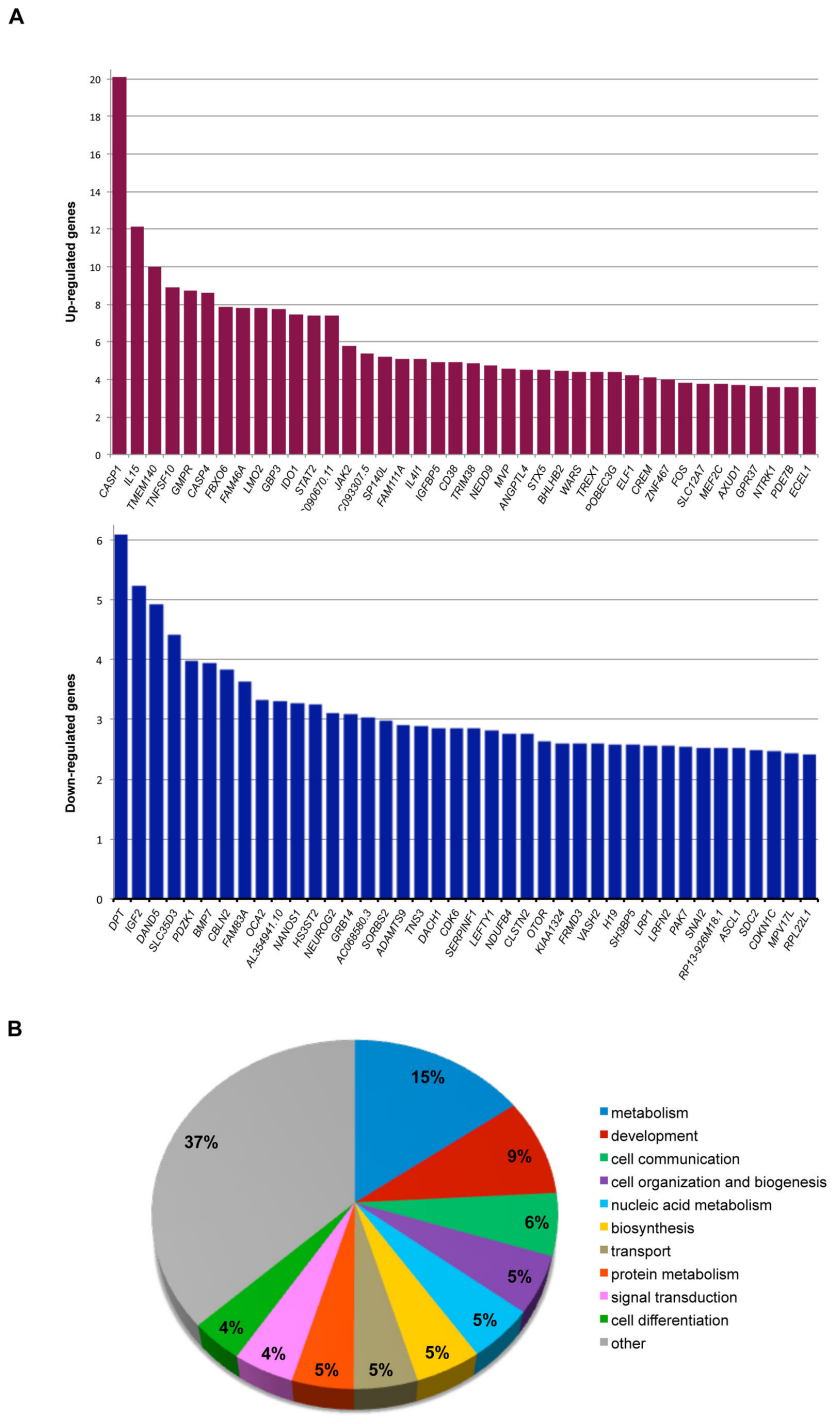
Overview of differentially expressed genes after *TCF4* knockdown. (A) Top 40 upregulated (upper panel) and downregulated (lower panel) genes after FDR correction (0.01) ranked by fold change. (B) Functional characterization of all differentially expressed genes (upregulated and downregulated) (FDR 0.01) using GOSLIM annotations. All annotation categories representing less than 4% of the gene list were grouped and labeled as “other”.

RNA prepared from siRNA-treated cells was converted to cDNA, labeled and hybridized to a Toray microarray for genome-wide transcript analysis. Two control groups, mock transfected and GAPDH siRNA transfected, were used for background correction. The GAPDH siRNA control was used because it has minimal off-target effects and is able to activate the RNA-induced silencing complex (RISC). Since TCF4 KD1 produced more gene expression changes than knockdown with TCF4 KD2 (overlap 79%) both treatment groups were combined in the ANOVA. A one-way ANOVA comparing the control (mock and GAPDH) and TCF4 (TCF4 KD1 and KD2) knockdown groups was used to identify differentially expressed genes in TCF4-depleted cells. Gene expression changes clustered with the experimental treatment indicating that each siRNA had a distinct but overlapping gene expression signature (Figure S1). A false discovery rate (FDR) of 0.01 was used to generate a high confidence list of 1204 differentially expressed genes (494 upregulated, 710 downregulated). The top 40 up- and downregulated genes ranked by fold change are illustrated in Figure 2A. Initial examination of the most robust gene expression changes in TCF4-knockdown cells (Figure 2A) appeared to suggest a role for TCF4 in apoptosis or inflammasome function (up-regulation of *CASP1* and *CASP4*), cell signaling (down-regulation of *IGF2*, *BMP7* and *LEFTY1*) and neurodevelopment (down-regulation of *NEUROG2*, *ASCL1* and *MEF2C*). Furthermore, several of the major gene expression changes in TCF4-knockdown cells involved transcription factors including ASCL1 and NEUROG2 that interact directly with TCF4 at E-boxes [6,10,11,25]. Finally, a number of imprinted genes, *IGF2*, *H19* and *CDKN1C* were prominent amongst the most significantly downregulated genes in TCF4-knockdown cells (Figure 2A).

qPCR was used to independently validate the gene expression changes detected on the microarray. We selected 5 up- (*FAS, NTRK1, CASP8, NOTCH1, CASP1*) and 5 down- regulated genes (*IGF2, CDKN1C, NEUROG2, BMP7, CDK6*) that had a fold change above or below 1.5. The genes were also selected on the basis of their known importance to developmental processes and their recurrent appearance in the downstream enrichment analyses (see below). Transcript abundance was measured from the same RNA samples used on the microarray, allowing a direct comparison of the inferred changes. Each of the 10 genes was confirmed as differentially expressed by qPCR and had similar fold changes to that detected in the microarray (Table 1).

**Table 1:** qPCR validation of differentially expressed genes.

	**Microarray**		**qPCR**	
**Gene**	Fold change	*P* value	Fold change	*P* value
*FAS*	1.9	2.88E-05	2.9	3.58E-05
*NTRK1*	3.8	1.53E-05	4.1	3.58E-05
*CASP8*	3.0	1.38E-05	10.4	2.40E-03
*NOTCH1*	2.3	1.02E-04	2.9	6.03E-05
*CASP1*	15.7	6.13E-05	9.8	8.06E-03
*IGF2*	-5.2	1.52E-06	-4.2	2.51E-05
*CDKN1C*	-2.4	2.36E-06	-2.3	1.02E-03
*NEUROG2*	-3.0	1.50E-05	-3.6	8.30E-07
*BMP7*	-3.7	1.34E-04	-2.4	5.46E-05
*CDK6*	-2.9	2.87E-07	-2.6	3.71E-06

Ten differentially expressed genes belonging to various functional categories were chosen for qPCR validation. All the genes selected for validation had similar fold changes by qPCR to that seen on the microarray. In both cases, the presented *P* values represent the statistical significance between the pooled control (mock treated, GAPDH KD) and TCF4 KD (KD1, KD2) groups.

### Enrichment analysis of differentially expressed genes in TCF4-depleted cells

Initial functional annotations associated with the 1204 differentially expressed genes in TCF4-knockdown cells were found to span a range of biological functions that cluster around the broad themes of metabolism, development and cell signaling (Figure 2B). To ascertain whether any biological functions were enriched in our high confidence gene list, we selected all genes with Entrez gene IDs for enrichment analysis. Of the 1204 genes that passed FDR correction of 0.01, 1031 had corresponding Entrez IDs (425 upregulated, 606 downregulated) and were statistically tested for GO annotation enrichment using Database for Annotation, Visualization and Integrated Discovery (DAVID) [26]. Functional annotations from up- and downregulated genes were compared separately to a background list of genes consisting of all expressed genes detected on the microarray. This analysis identified several biological processes that were significantly enriched (FDR 0.05) in TCF4 knockdown cells. These terms included non-coding RNA metabolic processes, apoptosis and regulation of NF-κB signaling (Table 2). Many of the terms in this analysis are related to apoptosis. Importantly, up- and down- regulated genes seemed to cluster by distinct functional annotations (Table 2). For example, regulation of the NF-κB and apoptotic signaling cascades and were significantly enriched in the upregulated genes whereas annotations relating to non-coding RNA metabolism, ribosome biogenesis and protein folding were downregulated as a consequence of TCF4 knockdown. Although not all the GO annotations listed in the downregulated gene category passed multiple test correction, they were nominally significant (*P* < 0.05) and functionally linked.

**Table 2:** GO term enrichment analysis using DAVID.

**GO annotation**		**Genes**	***P* value**	**Corrected** ***P* value**
**Upregulated genes (423 DAVID IDs)**				
1	GO:0043122~regulation of I-kappaB kinase/NF-kappaB cascade	*CD40, TFG, TNFSF10, CASP1, TBK1, PLK2, TRIM38, NOD1, RHOC, HTR2B, WLS, CARD8, SHISA5, TRADD, ZDHHC17*	4.87E-07	8.50E-04^*^
2	GO:0042981~regulation of apoptosis	*MEF2C, LOC100289713, NGFRAP1, ARHGEF6, NOTCH1, SH3GLB1, TNFSF13B, B4GALT1, TNFSF10, IDO1, BNIP3, APH1A, NOD1, ADAM17, CARD8, NGFR, SOCS3, KALRN, TRADD, SOX9, MSX2, AEN, CADM1, BARD1, CASP4, CREB1, CD38, CASP1, AKT1, CASP3, MCL1, JAK2, DPF2, TXNIP, FAS, TIA1, ANGPTL4, EYA1, YWHAZ, NTRK1, SIRT1, BCL2L13*	1.10E-05	1.92E-02^*^
3	GO:0009967~positive regulation of signal transduction	*CD40, BMPR1A, TFG, HIF1A, FKBP8, TNFSF10, CASP1, JAK2, TBK1, PLK2, TRIM38, NOD1, HTR2B, ADAM17, JAG1, RHOC, WLS, RICTOR, SMAD4, SHISA5, TRADD, ZDHHC17*	1.18E-05	2.06E-02^*^
**Downregulated genes (598 DAVID IDs)**				
1	GO:0034660~ncRNA metabolic process	*CARS2, FARSB, POP7, TSEN2, WDR12, EXOSC1, DUS3L, TARSL2, ADAT2, RPL35A, PDCD11, PIWIL1, IMP4, QTRTD1, TRMT10C, PUS3, EXOSC7, FTSJ1, MKI67IP, RPL7, DIMT1*	2.42E-05	4.14E-02^*^
2	GO:0022613~ribonucleoprotein complex biogenesis	*WDR12, GEMIN6, RRS1, EXOSC1, NCBP1, SURF6, RPL35A, TSR1, IMP4, PDCD11, GEMIN5, EXOSC7, FTSJ1, NUFIP1, MRTO4, RPL7, BYSL, DIMT1*	3.72E-05	6.37E-02
3	GO:0042254~ribosome biogenesis	*WDR12, RRS1, EXOSC1, SURF6, RPL35A, TSR1, IMP4, PDCD11, FTSJ1, EXOSC7, MRTO4, RPL7, BYSL, DIMT1*	8.01E-05	1.37E-01
4	GO:0006457~protein folding	*BAG2, DNAJC12, SEC63, RUVBL2, FKBP7, GRPEL1, APCS, SACS, PFDN6, HSPBP1, PPIF, PPID, CCT6A, URI1, PPIH*	7.06E-04	1.20

The Entrez Gene IDs of upregulated and downregulated genes were analyzed separately for biological process enrichment using DAVID. Enriched GO terms are ranked in order of decreasing significance. The gene IDs in each GO category are listed alongside the corrected (FDR) and uncorrected *P* values. For clarity redundant terms were removed. * Pass FDR correction of 0.05.

**Figure 3:**
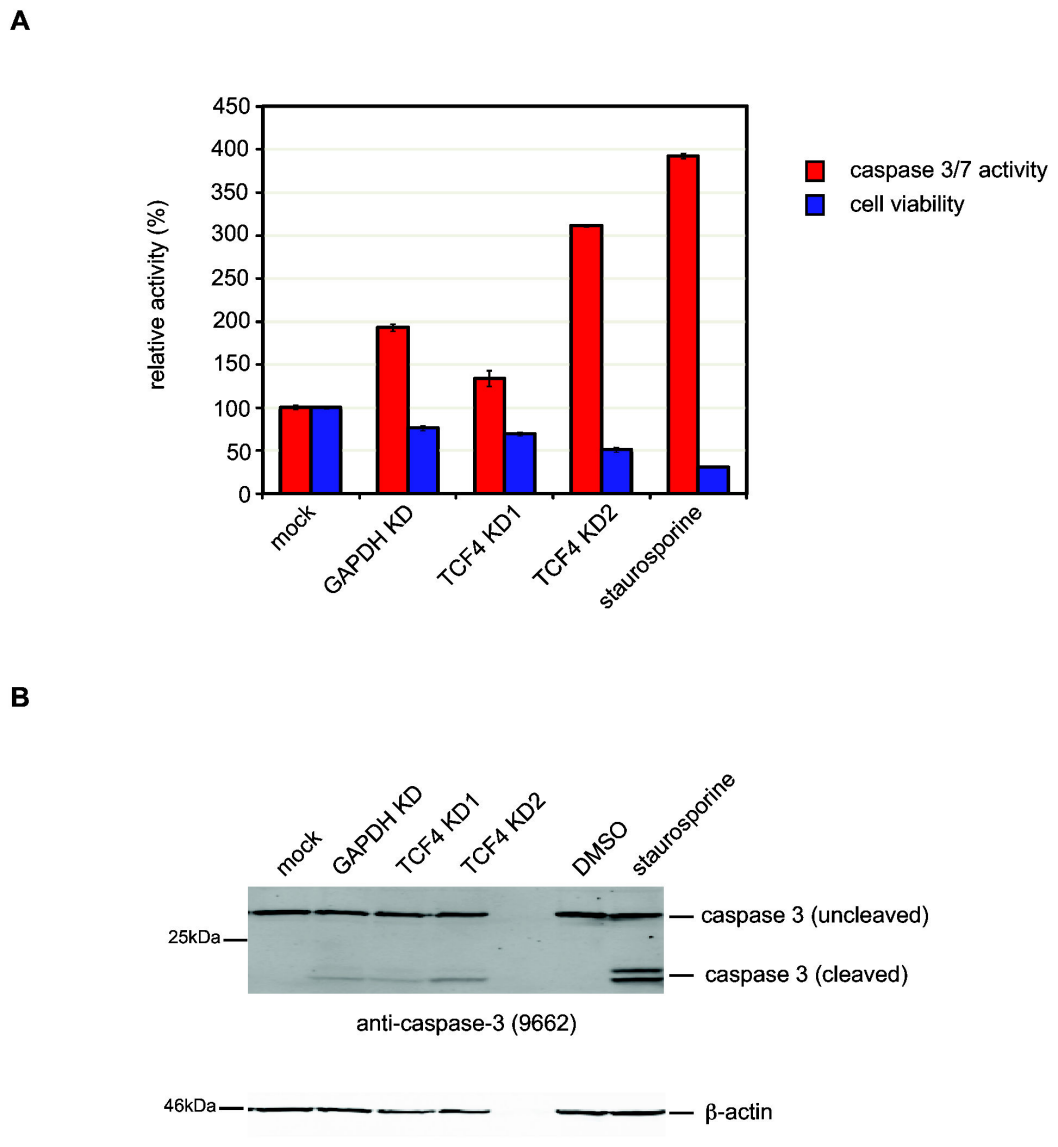
Knockdown of TCF4 induces apoptosis in SH-SY5Y cells. Cells were treated with siRNAs for 72h after which cell viability and caspase activity were measured (A). In addition to the siRNA treatment groups, untransfected SH-SY5Y cells were exposed to staurosporine (1µM in DMSO) and vehicle for 3h to induce apoptosis. Knockdown of TCF4 leads to a significant reduction in cell viability (*P* = 2.8x10^-16^, blue bars). Furthermore, TCF4-knockdown is also associated with an increase in caspase 3/7 activity compared to controls (*P* = 1.3x10^-3^, red bars). Although GAPDH knockdown is associated with reduced cell viability and elevated caspase 3/7 activity compared to mock-treated cells, both assays showed statistical significant differences between the control groups (mock and GAPDH) compared to TCF4-knockdown (TCF4 KD1 and KD2) supporting the microarray data. As expected, staurosporine treatment also reduced cell viability and increased caspase 3/7 activity in untransfected cells. Western blot analysis of caspase 3 processing after 72h knockdown shows that caspase 3 cleavage products are detected in siRNA treated cells (B). β-actin was used as a loading control for all treatment groups.

To determine whether the apoptotic cell death pathway was indeed activated in TCF4-depleted cells, we examined cell viability and caspase activation after treatment with the different siRNAs (Figure 3). TCF4-knockdown was associated with significantly reduced cell viability compared to the control groups (*P* = 2.8x10^-16^). Reduced cell viability was also associated with increased caspase 3/7 activity (*P* = 1.3x10^-3^) in TCF4-depleted cells (Figure 3A). Caspase-3 cleavage, which occurs in cells undergoing apoptosis, was also detected by western blotting in TCF4-depleted cells (Figure 3B). Interestingly, cells treated with TCF4 KD2 showed more evidence of apoptotic cell death and reduced viability compared to TCF4 KD1. In control experiments, acute staurosporine-treatment was associated with a robust increase in apoptotic cell death in untransfected SH-SY5Y cells. Although no gross differences in cellular morphology were evident between siRNA-treated cells and controls, condensed pyknotic nuclei were frequently observed in TCF4-depleted cells (Figure S2).

### Differential expression of genes in the TGF-β signaling and EMT pathways in TCF4-knockdown cells

To refine the analysis of downstream gene expression changes due to TCF4 knockdown, we utilized the MetaCore^TM^ (GeneGo) analytical suite which provides a manually curated database of “process networks” which detail more specific biological processes than GO annotations alone. In this analysis, the full list of up- and downregulated genes with Entrez IDs (1031 genes) were interrogated to reveal the concerted function of the top differentially expressed genes. This analysis identified a number of cellular processes in TCF4-knockdown cells including TGF-β signaling, epithelial to mesenchymal transition (EMT), hedgehog signaling, apoptosis and neurogenesis (Table 3). These data corroborate the findings using DAVID, as process networks relating to apoptosis (“Death Domain receptors and caspases in apoptosis” and “Apoptosis stimulation by external signals”) appeared in the list (Table 3).

**Table 3:** Process network enrichment in MetaCore.

**Category**	**Process Network**	**Genes**	***P* value**	**FDR**
Signal Transduction	TGF-β, GDF and Activin signaling	*PTPRK, PTGER2, CREB1, GATA3, CCND1, INHBA, SMAD4, FOS, SP1, SMAD2, HIF1A, ATF2, SMAD7, LEFTY1, MYC, TOB1, NOTCH1, CDK6*	3.54E-04	0.044^*^
Signal Transduction	BMP and GDF signaling	*CREB1, MSX2, BMPR1A, AKT1, BMP7, SMAD4, TLE1, ATF2, SMAD7, MYC, NODAL, SOX9, TOB1, CDK6*	8.02E-04	0.044^*^
Development	Regulation of epithelial-to-mesenchymal transition	*NOTCH1, CREB1, ACTA2, JAK2, BMP7, TRADD, SMAD4, FOS, SP1, SMAD2, HIF1A, ATF2, ABBP1, JAG1, SMAD7, ADAM17, SNAI2, IGF2, TJP1, SOX9*	8.38E-04	0.044^*^
Development	Regulation of telomere length	*TINF2, MAX, TEP1, SP1, HNRNPC, MYC, PTGES3, CDK6*	5.35E-03	0.190
Development	Hedgehog signaling	*NOTCH1, CREB1, HESX1, CCND1, SIRT1, INHBA, AKT1, BMP7, RBX1, SMAD4, ASCL1, ZIC1, SP1, ROCK2, PBX1. CDKN1C, HES1, JAG1, MYC, ADAM17, SOX9*	6.06E-03	0.190
Apoptosis	Death Domain receptors and caspases in apoptosis	*NGFRAP1, NGFR, TRADD, CD40, TIMP3, TNFSF10, CASP1, TRAF4, NOD1, PDCD5, CASP3, BIRC8, FAS, CASP4, CARD8*	7.26E-03	0.190
Apoptosis	Apoptosis stimulation by external signals	*NGFRAP1, JAK2, BID, NGFR, NTRK1, TRADD, SMAD4, TNFSF10, FOS, SMAD2, ADAM17, CASP3, FAS, BID*	9.66E-03	0.217
Development	Neurogenesis in general	*NOTCH1, CREB1, HESX1, GFRA3, INHBA, RCAN1, ASCL1, ZIC1, PBX1, NEUROG2, HES1, JAG1, ADAM17, CHRM3, MEF2C, SERPINI1*	1.24E-02	0.237
DNA damage	Checkpoint	*YWHAQ, CCND1, TLK2, BRIP1, CCND3, RUVBL2, ATF2, ATF3, RAD1, BARD1, MYC, YWHAZ, CDK6*	1.51E-02	0.237
Cardiac development	BMP and TGF-β signaling	*MSX2, ISL1, BMPR1A, BMP7, SMAD4, PDLIM3, MYH7, SMAD2, SMAD7, NODAL, SNAI2, SOX9, MEF2C*	1.51E-02	0.237

The Entrez gene IDs derived from the high-confidence list of 1031 differentially expressed genes in TCF4-knockdown cells were analyzed for enrichment using MetaCore^TM^. Each term is presented with its functional category, network and corresponding *P* value. * Pass FDR correction of 0.05.

Novel terms relating to particular signal transduction pathways and developmental processes were identified using MetaCore (Table 3). Specifically, three process networks passed stringent FDR correction (FDR 0.05). In the signal transduction category we identified process networks associated with “TGF-β, GDF and activin signaling” and “BMP and GDF signaling” to be over-represented. These terms refer to signaling through the TGF-β superfamily of ligands [27]. TGF-β ligands operate through the activation of transmembrane serine–threonine receptor kinases that phosphorylate SMAD proteins to coordinate cell-type specific gene expression. MetaCore analysis revealed that several elements of the TGF-β signaling pathway where differentially expressed after TCF4 knockdown. Differentially expressed genes included two ligands of the TGF-β family (INHBA and BMP7), the BMPR1A receptor, and several of the SMAD proteins (SMAD2, SMAD4, SMAD7), demonstrating that each level of the pathway was affected (Table 4).

**Table 4:** Gene expression changes associated with TGF-β and Notch signaling pathways in TCF4-knockdown cells.

**Gene**	**FC**	***P* value**	**Description**
**TGF-β signaling pathway**			
*BMP7*	-3.94	1.34E-04	BMP Ligand
*BMPR1A*	2.46	1.81E-05	Tyrosine kinase receptor (BMP)
*SMAD2*	-2.15	4.38E-04	R-SMAD (TGF/Nodal)
*SMAD4*	1.49	4.71E-04	co-SMAD
*SMAD7*	1.38	3.97E-04	I-SMAD
*SMAD6*	*1.23*	1.04E-02^*^	I-SMAD
*SMAD1*	*1.28*	9.59E-03^*^	R-SMAD (BMPR1)
*NODAL*	-1.25	2.27E-04	Ligand (Nodal)
*INHBA*	-2.38	2.68E-07	Ligand (Activin A)
*LEFTY1*	-2.81	3.37E-04	Ligand (Inhibitor of Nodal)
**Notch signaling pathway**			
*NOTCH1*	2.40	1.02E-04	Ligand receptor
*NOTCH2*	*-1.43*	8.48E-03^*^	Ligand receptor
*JAG1*	2.59	4.61E-04	Ligand receptor (inhibitor of Notch)
*ADAM17*	1.44	3.83E-04	metallopeptidase (Notch-cleavage)
*APH1A*	1.64	1.57E-04	γ-secretase complex (Notch-cleavage)
*HES1*	1.65	4.67E-05	Notch effector
*HES7*	*-1.54*	6.40E-03^*^	Notch effector

Gene expression changes in manually curated signaling pathways in TCF4-knockdown cells. The data are presented as statistically significant (FDR 0.01) gene expression changes (fold change) for the genes in the TGF-β and Notch signaling pathways. * Pass FDR correction of 0.05.

The third term that remained statistically significant after FDR correction was in the development category and relates to EMT (Table 3). EMT is a developmental process whereby cells loose their adhesive properties and become more motile. EMT is essential for neural tube formation and is thought to be a key step regulating cancer cell metastasis [28]. Two important EMT regulators, *SNAI2* and *DEC1* (*BHLHB2* in Figure 2A) are differentially expressed in response to TCF4 depletion. *SNAI2* promotes EMT and is downregulated in TCF4-knockdown cells whereas *DEC1* is upregulated in TCF4-knockdown cells (Figure 2A). It is also noteworthy that differentially expressed genes associated to the EMT category are also of general importance to development as they include elements of the Notch, BMP and IGF signaling pathways.

RT-PCR was used to gain further support for the differential expression of EMT genes in TCF4-depleted cells (Figure S3). These data confirm the gene expression changes detected on the microarray and show that in addition to *SNAI2*, *SNA11* is also downregulated in TCF4-depleted cells. Importantly, expression of the closely related class I bHLH gene *TCF3/E47* was unaltered in TCF4-depleted cells suggesting the alterations in the EMT gene expression pathway occurred independently of E47.

The IGF-signaling pathway also appears to be altered in TCF4-depleted SH-SY5Y cells [29]. Specifically, *IGF2* and *GRB14* are among the most significantly downregulated genes whereas *IGFBP5* and the genes encoding other IGF-binding proteins are upregulated in our dataset (Figure 2 and Table 5). These alterations in gene expression are also evident in downstream elements of the IGF-signaling pathway and include down-regulation of several kinases and adaptor molecules such as *AKT1* and *RPS6KB1* that encodes the p70 ribosomal S6 kinase (Table 5).

**Table 5:** Gene expression changes associated with the IGF signaling pathway in TCF4-knockdown cells.

**Gene**	**Fold change**	***P* value**	**Description**
*IGF2*	-5.22235	1.52E-06	IGF ligand
*IGFBP5*	4.90659	2.35E-05	IGF carrier protein
*IGFBP3*	1.28834	1.17E-02^*^	IGF carrier protein
*IGFBP4*	2.59348	3.09E-03^*^	IGF carrier protein
*IGFBPL1*	-1.43755	8.52E-05	
*GRB14*	-3.08126	1.79E-05	Receptor-bound protein (inhibitor)
*GRB7*	-1.22578	1.95E-04	Receptor-bound protein (inhibitor)
*PIK3CG*	-1.75377	9.67E-03^*^	Signal transduction (kinase)
*PIK3CA*	1.58222	6.16E-04^*^	Signal transduction (kinase)
*PIK3C2B*	-1.45293	5.42E-03^*^	Signal transduction (kinase)
*PIK3CD*	-1.30542	7.37E-03^*^	Signal transduction (kinase)
*PDPK1 (PDK1)*	-1.98747	8.88E-05	AKT inhibition (kinase)
*AKT1*	1.36045	3.53E-04	Signal transduction (kinase)
*YWHAG*	-1.9787	7.64E-05	Regulatory co-factor
*YWHAZ*	1.2674	3.63E-04	Regulatory co-factor
*FOXO1A*	1.6064	3.75E-03^*^	Transcription factor
*FOXO3B*	-1.72656	3.65E-03^*^	Transcription factor
*RPS6KB1 (S6K1)*	-1.2898	3.83E-03^*^	Ribosomal subunit kinase
*RPS6KC1*	1.78394	4.15E-03^*^	Ribosomal subunit kinase
*RPS6KA3*	1.47367	6.83E-03^*^	Ribosomal subunit kinase
*RPS6KA1*	-1.32884	2.57E-03^*^	Ribosomal subunit kinase

Gene expression changes in the manually curated IGF signaling pathway in TCF4-knockdown cells. The data are presented as statistically significant (FDR 0.01) gene expression changes sorted by fold change for the genes in the IGF signaling pathway [29]. * Pass FDR correction of 0.05.

### Altered expression of neurogenic and neurological disease genes in TCF4-depleted cells

One of the initial aims of this study was to identify gene expression changes in neurodevelopmental genes. Accordingly, we found that the proneural genes *ASCL1* and *NEUROG2* were downregulated in TCF4-depleted cells (Figure 2A). In addition to the proneural genes, a number of genes implicated in rare Mendelian forms of mental retardation were differentially expressed in TCF4 knockdown cells (Table 6). Interestingly, these differentially expressed genes are mutated in disorders that resemble Rett syndrome and have phenotypic similarities to PTHS. For example, *MEF2C* is found in the top 40 most significantly upregulated genes in TCF4-knockdown cells (Figure 2A). Furthermore, *UBE3A* (mutated in AS) is downregulated in TCF4 knockdown cells whereas the MWS gene and EMT regulator *ZEB2*, is upregulated (Table 6).

**Table 6:** Altered expression of several mental retardation (MR) genes in TCF4-knockdown cells.

**Gene**	**Disease**	**OMIM**	**Inheritance**	**FC**	***P* value**	**Reference**
*TCF4*	PTHS	610954	AD	-3.10	2.02 E-05	[9]
*MEF2C*	Syndromic MR	613443	AD	3.73	1.37 E-05	[50]
*UBE3A*	AS	105830	imprinted	-1.39	1.56 E-03^*^	[59]
*ZEB2*	MWS	235730	AD	1.48	3.60 E-03^*^	[60]
*FMR1*	Fragile X syndrome	300624	X-linked	1.94	1.51 E-02^**^	[61]

Data presented are the fold change and the corrected *P* value (FDR 0.01) derived from the microarray. *Pass FDR correction of 0.05, **pass FDR correction of 0.1. Abbreviations: PTHS, Pitt-Hopkins syndrome; AS, Angelman Syndrome; MWS, Mowat-Wilson syndrome.

## Discussion

It is now well established that rare, highly penetrant *TCF4* alleles are associated with neurodevelopmental phenotypes whereas common variants are associated with disorders such as schizophrenia [6–8,13,14]. Recent cognitive and imaging studies have also shown that TCF4 is important for normal brain function [1,15]. However, knowledge of TCF4-regulated genes and pathways in the brain is comparatively sparse. Using genome-wide transcriptional profiling of SH-SY5Y cells where TCF4 was knocked down with siRNAs, we demonstrate that TCF4 reduction affects the expression of genes involved in cell signaling, cell survival and neurodevelopment. By deconstructing the top gene expression changes, we also show that genes controlling apoptosis tend to be upregulated whilst genes that support processes involving non-coding RNA metabolism are downregulated. Process network enrichment analysis also suggests that the TGF-β, Notch and IGF signaling pathways may converge on the EMT pathway in TCF4-depleted cells.

### GO enrichment analysis implicates TCF4 in apoptosis and non-coding RNA metabolism

We used several bioinformatics tools to search for processes and pathways that may be altered in TCF4-depleted cells. Initial results from GO term enrichment analysis using DAVID showed that apoptosis and NF-κB signaling were statistically significant processes among the upregulated genes. Shared terms in GO categories between NF-κB signaling and cell death categories show that many of the genes in each pathway are the same, indicating some degree of convergence between NF-κB signaling and apoptosis (Table 2). In neurons, NF-κB regulates the expression of genes participating in seemingly diverse aspects of neurodevelopment, learning and memory [30]. NF-κB signaling has also been implicated in enhancing neuronal apoptosis associated with neurodegenerative disease, brain injury and inflammatory conditions [31]. Similarly in neurons, caspase activation is known to be important for axon pruning and synapse elimination and can mediate some of the chronic neuropathological events associated with brain injury or neurodegeneration [32]. Since acute knockdown of TCF4 over a period of 72h reduced cell viability and increased caspase 3/7 activity, up-regulation of some caspases and components of the NF-κB pathway in TCF4-depleted cells may induce cell death (Figure 3). It is therefore possible that TCF4 may regulate the expression of some caspase genes and other components of the pro-apoptotic signaling pathway in SH-SY5Y cells.

### TCF4-knockdown affects genes in the TGF-β signaling pathway

To gain further insight in to TCF4-regulated pathways, we used MetaCore to identify network processes that were altered in TCF4-depleted cells. Enrichment analysis on the top 1031 differentially expressed genes, demonstrated that several components of the TGF-β signaling pathway are affected. Specifically, we found robust down-regulation of the TGF-β superfamily ligands (*BMP7*, *NODAL*, *LEFTY1* and *INHBA*) and altered expression of several downstream components of the TGF-β signaling cascade including *BMPR1A*, and some of the SMAD transcription factors (Table 4). TGF-β signaling regulates many aspects of cell proliferation, differentiation, migration and apoptosis [27]. In the nervous system, TGF-β signaling regulates neural crest formation and in also required for neurogenesis, neurite outgrowth and synaptogenesis [33,34]. Interestingly, *Bmp7* regulates the survival and proliferation of neural progenitor cells in the developing neocortex of mice and maintains *Ngn2* (the murine orthologue of *NEUROG2*) in ventricular and subventricular zones of the cortex [35]. Notably, both *BMP7* (FC, -3.93) and *NEUROG2* (FC, -3.10) are robustly downregulated in TCF4-depleted cells suggesting that TCF4 may coordinately regulate each gene (Figure 2A).

### TCF4-knockdown affects EMT regulators

In addition to driving developmental programs, TGF-β signaling is also involved in EMT.

Process network enrichment identified genes in the EMT pathway to be differentially expressed in TCF4-knockdown cells compared to controls (Table 4). This pathway governs the transition between epithelial and mesenchymal phenotypes known to be important during development and in cancer metastasis [36]. TGF-β activates SMADs 3 and 4 to transcribe *SNAI1* that represses expression of critical epithelial cell genes such as *CDH1* (E-cadherin, a commonly used molecular marker of the epithelial phenotype) allowing activated cells to switch to the mesenchymal phenotype [27]. In addition to components of the TGF-β signaling pathway, the EMT regulators *SNAI1/2* and *DEC1* (*BHLHB2* on the array) were differentially expressed in TCF4-knockdown cells (Figure 2A and Figure S3). SNAI1 and 2 are transcriptional repressors that bind to E-boxes in the promoter regions of genes including *Cdh1* [37]. Similarly, DEC1 is another transcriptional repressor that regulates EMT in pancreatic cancer cells in response to TGF-β stimulation [38]. Our data are therefore consistent with findings in other cell types showing that the E-proteins TCF4 and E47 are involved in EMT [39–41].

### Gene expression changes in the IGF signaling pathway

In addition to changes in gene expression associated with the TGF-β and Nodal signaling, TCF4-knockdown also alters the expression of components of the IGF signaling pathways (Table 5). TCF4-knockdown is associated with a 5.2 fold down-regulation of *IGF2* and up-regulation of the genes encoding IGF binding proteins, IGFBP3, -4 and -5 (Figure 2A and Table 5). In the brain, IGF2 is required for memory consolidation and enhancement [42], adult hippocampal neurogenesis [43], synapse formation and dendritic spine maturation [44]. Since *IGF2* has a role in learning and memory and PTHS patients have profound intellectual disability [45], TCF4 regulation of *IGF2* expression may be a determinant of cognitive dysfunction. IGF1 treatment has been shown to reverse some of the neurophysiological abnormalities in a mouse model of Rett Syndrome lacking methyl CpG-binding protein 2 (*Mecp2* [46]). Because IGF1 and IGF2 activate the same receptor, IGF2 may have some utility in reversing some of the cognitive deficits in PTHS patients. Another component of the IGF pathway *RPS6KB1* (encoding a member of the ribosomal protein S6 kinase family), is also downregulated in TCF4-depleted cells (Table 5). *RPS6KB1* may be particularly important in the context of neurodevelopment because genetic ablation of this gene rescues multiple physiological and behavioural phenotypes in a mouse model of fragile X syndrome, caused by aberrant synaptic translation [47].

### Altered expression of neurodevelopmental genes in TCF4-knockdown cells

*ASCL1* and *NEUROG2* are important neurogenic bHLH transcription factors that interact directly with TCF4 and appear among the top downregulated genes in TCF-depleted cells (Figure 2A and Table 2 respectively). Since both of these proneural genes are downregulated, this may indicate that as well as regulating proneural activity through protein: protein interactions, TCF4 may also regulate proneural gene expression. In addition to *ASCL1* and *NEUROG2*, other neurodevelopmental transcriptional regulators such as, *MEF2C* (syndromic mental retardation) and *ZEB2* (MWS) were also differentially expressed in TCF4-depleted cells.

PTHS, MWS and AS are sometimes classified as Rett-like syndromes because of their similar clinical presentation and genetics [9,48]. Furthermore, haploinsufficiency of *MEF2C* also results in a form of severe mental retardation, with absent speech, hypotonia and epilepsy [49,50]. Importantly, we found that the genes for each of these phenotypically similar disorders (*MEF2C*, *ZEB2* and *UBE3A*) were differentially expressed in TCF4-depleted cells (Table 6). From a mechanistic perspective, TCF4, ZEB2 and MEF2C can all regulate gene expression at E-boxes in the promoter regions of certain genes [51,52]. These data, allied with the phenotypic overlap between these disorders, suggest that each of these genes may participate in a similar neurodevelopmental transcriptional pathway. Thus, alterations in the activity or levels of TCF4 as seen in PTHS, autism and possibly schizophrenia, may be associated with dysregulation of several transcription factors that control neurodevelopmental gene expression programs at E-box containing promoters.

In summary, microarray analysis of SH-SY5Y cells where TCF4 has been acutely knocked down with siRNAs identifies gene expression changes in pathways associated with apoptosis, signaling, EMT and neurodevelopment. These data provide an important insight in to the cellular processes that are regulated by TCF4. Our data also suggest a novel shared mechanism in a subset of neurodevelopmental disorders that regulate aspects of normal brain function and may also contribute to the molecular pathology of common diseases such as autism and schizophrenia.

## Methods

### RNA interference and transfection

27 mer RNA oligonucleotide duplexes were designed using predictive algorithms (siDESIGN Center, http://www.thermoscientificbio.com/design-center/; siMAX^TM^ Design Tool, http://www.eurofinsdna.com) and were selected for their predicted efficacy scores and homology to regions of interest. Two duplexes (KD1 (exon 12), 5’-GGGACAGACAGUAUAAUGGCAAAUAGA; KD2 (exon19), 5’-AUAAUGACGAUGAGGACCUGACACCAG) were designed to target constitutive exons found in all the published human *TCF4* transcripts [19]. The GAPDH siRNA 5’- CGGAGUCAACGGAUUUGGUCGUAUUGG was used in control experiments to identify transcripts that were altered by RNA interference having minimal off-target affects. siRNAs (Eurofins MWG Operon) were stored as a 50mM stock in siMAX Universal Buffer (30mM HEPES, 100mM KCl, 1mM MgCl_2_; pH 7.3) at -80°C.

SH-SY5Y (American Type Culture Collection, ATCC) cells were cultured in Dulbecco’s modified Eagle medium (DMEM, Invitrogen) supplemented with 10% (v/v) foetal calf serum (FCS, PAA laboratories) without antibiotics. 24h after seeding, cells were transfected with the different siRNA duplexes (TCF4 KD1 and KD2, GAPDH or mock) using Lipofectamine® RNAiMAX (Invitrogen) at a final concentration of 10nM. 48h after the first transfection the cells were re-transfected using the same conditions. The following day (72h after the first transfection), RNA and protein were prepared from each experimental condition. Each transfection was performed in quadruplicate and three biological replicates were used for gene expression analysis whilst the remaining sample was used for western blotting.

### Western blotting

For quantitation of protein levels after siRNA treatment, transfected SH-SY5Y cells were lysed in RIPA buffer and the resulting lysates were clarified by centrifugation. 10µg of each lysate was separated on a 12% gel polyacrylamide gel and transferred to nitrocellulose as described previously [53]. Membranes were blocked overnight in blocking buffer (5% (w/v) milk powder in Tris-buffered saline containing Tween-20 (TBST)) and then incubated with anti-TCF4 (Abnova) and anti-α-tubulin antibodies (12G10, Developmental Studies Hybridoma Bank, The University of Iowa) diluted in blocking buffer. After extensive washing in TBST, blots were incubated with the appropriate secondary antibody prior to imaging on a LI-COR Odyssey infrared scanner. Proteins levels were quantified using the Odyssey software version 2.1 as described previously [54,55].

### Quantitative and reverse transcriptase PCR (qPCR and RT-PCR)

RNA was extracted from siRNA-treated cells using RNeasy Plus Mini Kits (QIAGEN) according to the manufacturer’s instructions. Total RNA was treated with Ambion® TURBO DNA-free™ DNase I (Invitrogen) before being converted to cDNA using the ProtoScript® M-MuLV First Strand cDNA Synthesis Kit (NEB). Triplicate 25µl qPCR reactions were prepared on a Corbett robot (Corbett Robotics CAS1200/QIAGEN) using 10ng of cDNA as a template, SensiMix^TM^ SYBR No-ROX (Bioline) and an optimized concentration of gene-specific primers. qPCR was then performed on a Qiagen Rotor-Gene 6000 (Corbett Robotics/QIAGEN). GAPDH, TCF4 and 18S primer efficiencies were all optimized prior to use on experimental samples. Gene expression was quantified using the ΔCT method and 18S rRNA as an internal control. Primer sequences are listed in Table S1A.

TaqMan® gene expression assays were used to validate gene expression changes (*NOTCH1, FAS, CASP8, CASP1, NTRK1, NEUROG2, CDK6, BMP7, IGF2 and CDKN1C*) after knockdown using pre-optimized reagents (Table S1B). Briefly, 1-10ng of cDNA was used as template in 20µl reactions with the TaqMan® Fast Advanced Master Mix depending on transcript abundance (Applied Biosystems/Life technologies). All samples were run in triplicate according to the Fast Advanced Master Mix protocol using the ABI 7900HT Fast Real-Time PCR System (Applied Biosystems/Life technologies). Results were then analyzed on the RQ Manager 1.2 (Applied Biosystems/Life technologies). All genes were normalized to 18S rRNA using the ΔCT method. To calculate fold change, the relative transcript abundance from mock and GAPDH KD samples were pooled and compared to the values of pooled KD1 and KD2 samples. A two-tailed t-test was performed on these two groups to determine statistical significance.

For semi-quantitative RT-PCR, 10ng of cDNA was amplified (30 cycles) in a 25µl reaction with REDTaq® DNA Polymerase according to manufacturer’s instructions (Sigma). The sequences of the gene-specific primer pairs are listed in Table S1C. 20µl of the PCR reaction was separated on a 2.5% agarose gel containing ethidium bromide. PCR products were imaged with a GelDoc-It® TS Imaging System (UVP).

### Toray Microarray

Three biological replicates from four treatment groups (TCF4 KD1and KD2, GAPDH KD and mock) were sent for microarray analysis (Central Biotechnology Services, School of Medicine, Cardiff University). Briefly, the RNA concentration and quality was assessed on a RNA Nano Chip using the Agilent 2100 Bioanalyser (Agilent Technologies). RNA was labeled, fragmented then hybridized to 3D-gene Human Oligo chip 25 k (Toray Industries Inc., Japan). Chips were scanned three times at different intensities and normalized. The resulting signal intensity data was then analyzed in-house. Data was imported into Partek Genomics Suite 6.6 (Partek Inc.) and analyzed. As there were only three replicates in each group, probe sets with more than one missing value in any group were removed from the analysis. A one-way ANOVA comparing control (mock and GAPDH) and TCF4 knockdown (KD1, KD2) groups was then performed to identify differentially expressed genes. A false discovery rate (FDR) was used to correct for multiple testing (FDR < 0.01, 1204 differentially expressed genes; FDR < 0.05, 5374 differentially expressed genes). The data discussed in this publication have been deposited in NCBI’s Gene Expression Omnibus [56] and are accessible through GEO Series accession number GSE48367 (http://www.ncbi.nlm.nih.gov/geo/query/acc.cgi?acc=GSE48367).

### Enrichment Analysis

The Entrez Gene IDs from the high confidence (FDR 0.01, 1031 IDs) gene list was used as the initial dataset for enrichment analysis. In addition, a background list consisting of Entrez Gene IDs from all genes detected by the microarray was also generated (18966 IDs). Biological process GO term enrichment (GOTERM_BP_FAT) was performed using DAVID v 6.7 [26]. 425 upregulated and 606 downregulated genes were analyzed separately using the same background list. Process Network term enrichment was performed using MetaCore^TM^ (GeneGo, Thomson Reuters). Here, the full FDR corrected list (1031 IDs) was uploaded and compared to the background list of genes. An FDR cut off of 0.05 was used to determine significant enrichment from both analyses. Manually curated pathways were annotated using the Kyoto Encyclopedia of Genes and Genomes (KEGG, http://www.genome.jp/kegg/) Pathways and WikiGenes (http://www.wikigenes.org) web resources [57,58]. Please note that we have used the HGNC (Human Genome Nomenclature Committee) nomenclature for each gene but provide common synonyms for some of the genes that were followed up in this study.

### Cell viability and caspase assays

SH-SY5Y cells were plated in 96 well plates and treated with the appropriate siRNAs for 72h as described above. Cell viability and caspase 3/7 activation were measured in the same plate using the CellTiter-Fluor™ Cell Viability (Promega) and the Caspase-Glo® 3/7 (Promega) assays. Staurosporine-treated (1µM in DMSO, 3h at 37^°^C) SH-SY5Y cells were used as a positive control for each assay. Cell viability was measured in 24 replicates of each condition whereas 12 biological replicates were used to assay caspase 3/7 activity. The data were normalized to the levels in the mock-transfected cells, nominally 100%, and represented as the mean and standard error. T-tests were used to compare treatment groups. In addition to the microplate assays, caspase 3 cleavage was assessed by western blotting. After treatment, dead cells were collected from the media by centrifugation and lysed with the remaining live cells. 15μg of each lysate was used for western blotting with a caspase 3 (#9662, Cell Signaling) and β-actin (AC-15, Sigma) antibodies.

### Immunocytochemistry

SH-SY5Y cells were processed for immunocytochemistry as described previously [53,54]. Briefly, siRNA-treated cells grown on coverslips were fixed in 4% (v/v) formaldehyde, permeabilised with 0.01% (v/v) Triton-X100 and blocked with 10% (v/v) foetal calf serum. Cells were labeled at room temperature with Alexa Fluor 488 phalloidin (165nM, Invitrogen), Hoechst (1µg/ml, Invitrogen) and an anti-TCF4 polyclonal antibody (Covalab, UK) for 1h followed by Alexa Fluor 546 goat anti-rabbit IgG (Invitrogen) for a further 30min. After washing in PBS, coverslips were mounted in Aqua-Poly/Mount (Polysciences, Inc.) and images were captured on a Leica SP2 confocal microscope using a 63X oil immersion objective lens. Images were assembled using Adobe Photoshop CS4 and only adjusted for brightness and contrast.

## Supporting Information

Figure S1 **Hierarchical clustering of the top differentially expressed genes.** The top 1204 differentially expressed genes (FDR 0.01) cluster by treatment group as evident from the dendrogram to left of the heatmap. Samples from the control treatments (mock and GAPDH KD) and TCF4 KD treatment (KD1 and KD2) are shown to cluster into defined groups (white and black respectively). The top section of the heat map displays differential expression relative to controls: up-regulated genes are shown in red while down-regulated genes are coloured blue.
(EPS)

Figure S2 **Cellular imaging of siRNA-treated SH-SY5Y cells.** SH-SY5Y cells were treated with siRNAs for 72h as described above. Fixed cells were stained with Alexa Fluor 488 phalloidin (F-actin), Hoescht-33342 (nuclei) and with an anti-TCF4 polyclonal antibody. Although there is a marked reduction in TCF4 immunoreactivity in TCF4 KD cells, no apparent morphological differences are observed between treatment groups after 3 days knockdown. A few condensed pyknotic nuclei, indicative of apoptosis were observed after TCF4 KD treatment (arrow heads).
(TIF)

Figure S3 **Semi-quantitative RT-PCR analysis of EMT-regulating transcription factors in TCF4-knockdown cells.** RT-PCR was used to confirm differential expression of several transcription factors that drive EMT. *SNAI1* and *SNAI2* transcripts are down-regulated in TCF4-knockdown cells whereas *BHLHE40* (*DEC1*) and *MEF2C* transcripts are up-regulated. These data also show that *TCF3/E47* transcripts, which encode a paralogue of TCF4 that is required for EMT, are unaltered in TCF4-depleted cells. Note that alterations in *SNAI1* were not evident on the microarray possibly due to its low expression in SH-SY5Y cells.
(TIF)

Table S1 **Primer sequences and TaqMan probes.**
(A) qPCR primers used for knockdown quantification (SYBR Green).
(B) TaqMan probe IDs used for microarray validation.
(C) Primers used for semi-quantitative RT-PCR.
(DOCX)
