# Supplementary figures and images for: Knockdown of Human TCF4 Affects Multiple Signaling Pathways Involved in Cell Survival, Epithelial to Mesenchymal Transition and Neuronal Differentiation
Source: PLoS One. 2013 Aug 23;8(8):e73169. doi: 10.1371/journal.pone.0073169 (PMC3751932; doi:10.1371/journal.pone.0073169)

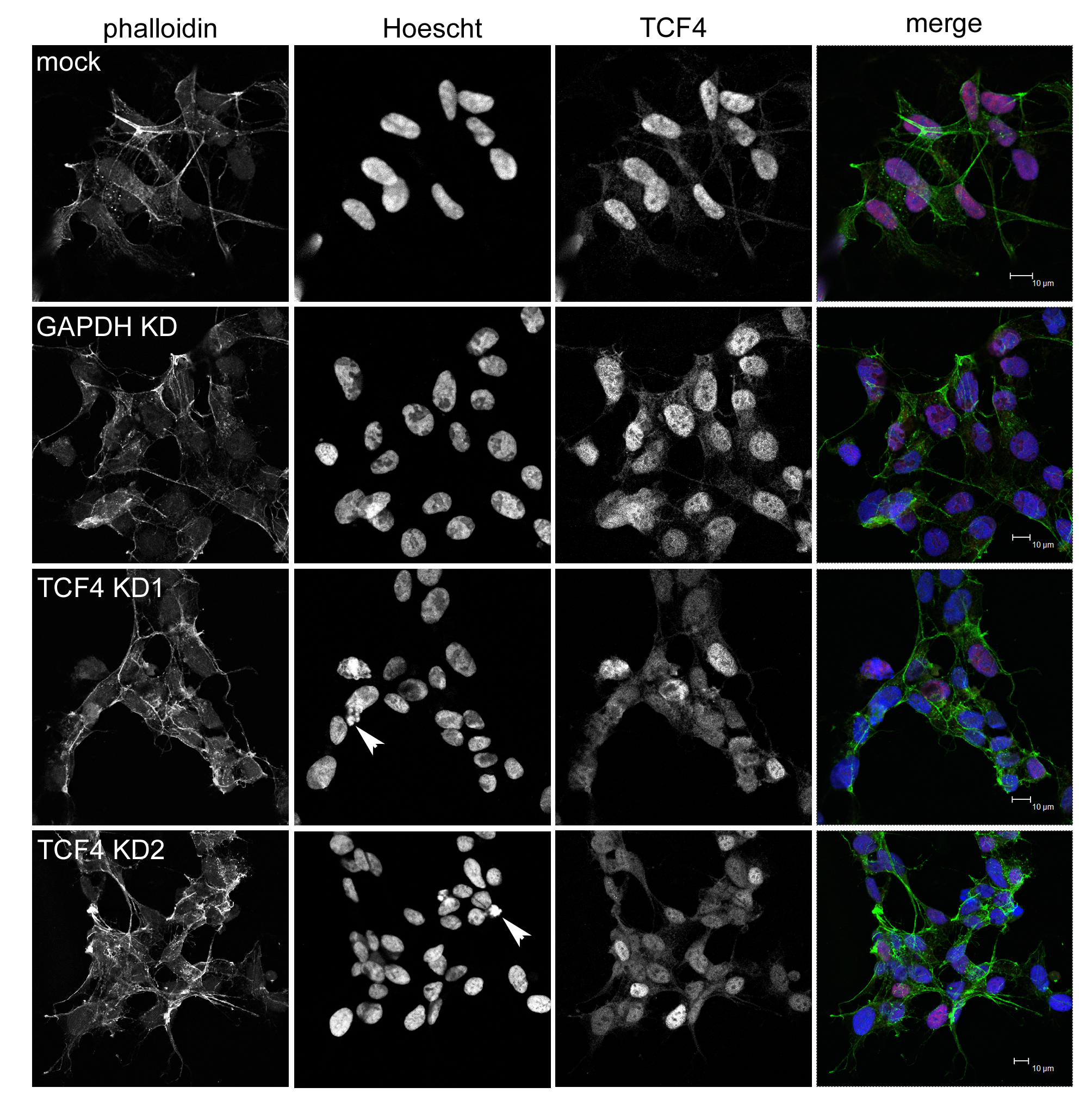

Supplement: Figure S2 — Cellular imaging of siRNA-treated SH-SY5Y cells. SH-SY5Y cells were treated with siRNAs for 72h as described above. Fixed cells were stained with Alexa Fluor 488 phalloidin (F-actin), Hoescht-33342 (nuclei) and with an anti-TCF4 polyclonal antibody. Although there is a marked reduction in TCF4 immunoreactivity in TCF4 KD cells, no apparent morphological differences are observed between treatment groups after 3 days knockdown. A few condensed pyknotic nuclei, indicative of apoptosis were observed after TCF4 KD treatment (arrow heads). (TIF) [file pone.0073169.s002.tif]

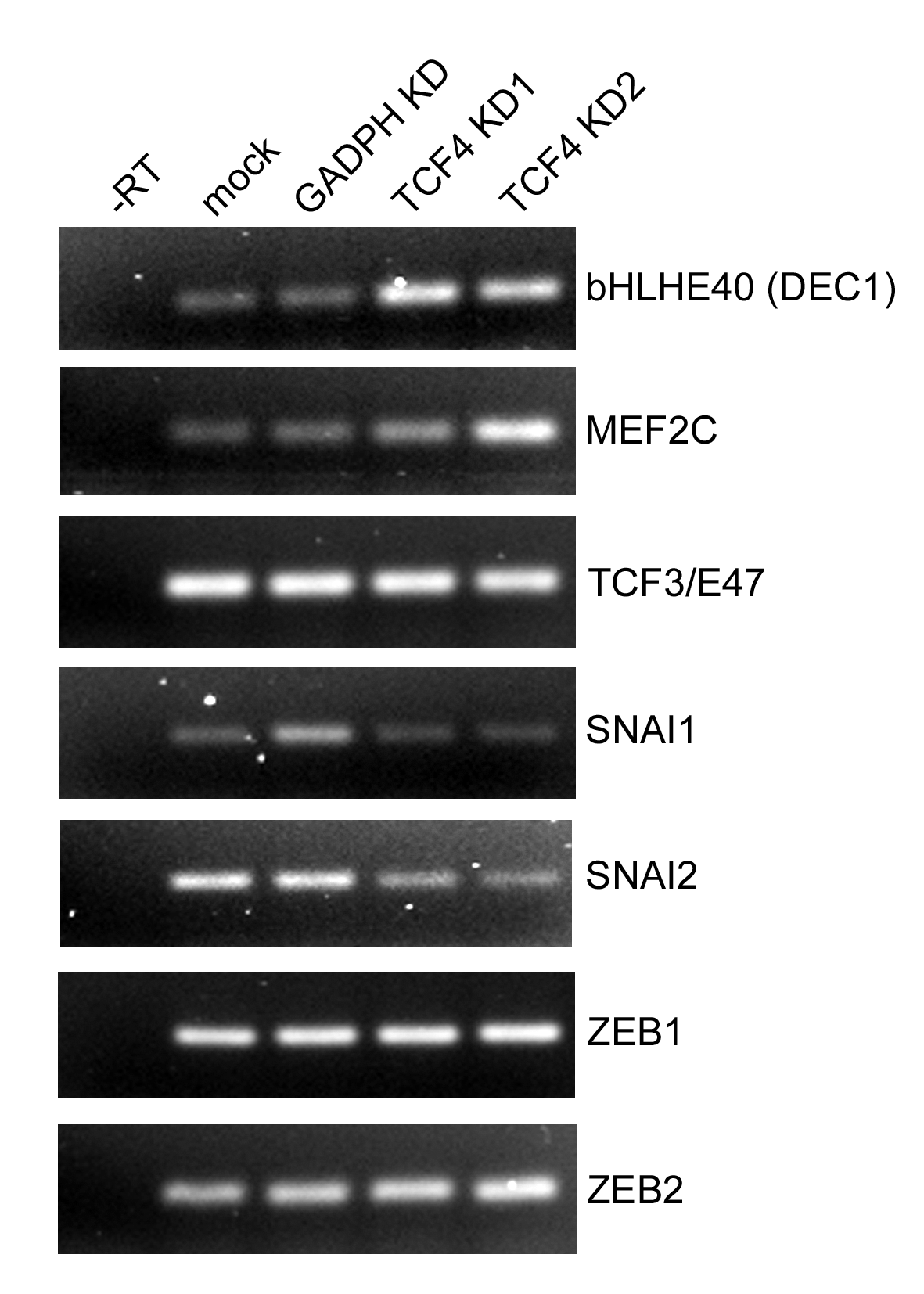

Supplement: Figure S3 — Semi-quantitative RT-PCR analysis of EMT-regulating transcription factors in TCF4-knockdown cells. RT-PCR was used to confirm differential expression of several transcription factors that drive EMT. SNAI1 and SNAI2 transcripts are down-regulated in TCF4-knockdown cells whereas BHLHE40 (DEC1) and MEF2C transcripts are up-regulated. These data also show that TCF3/E47 transcripts, which encode a paralogue of TCF4 that is required for EMT, are unaltered in TCF4-depleted cells. Note that alterations in SNAI1 were not evident on the microarray possibly due to its low expression in SH-SY5Y cells. (TIF) [file pone.0073169.s003.tif]
