# Supplementary material for: Knockdown of Human TCF4 Affects Multiple Signaling Pathways Involved in Cell Survival, Epithelial to Mesenchymal Transition and Neuronal Differentiation
Source: PLoS One. 2013 Aug 23;8(8):e73169. doi: 10.1371/journal.pone.0073169 (PMC3751932; doi:10.1371/journal.pone.0073169)
Supplement: Table S1 — Primer sequences and TaqMan probes. (A) qPCR primers used for knockdown quantification (SYBR Green). (B) TaqMan probe IDs used for microarray validation. (C) Primers used for semi-quantitative RT-PCR. (DOCX) [file pone.0073169.s004.docx]

**Table S1A**

| **Gene Name** | **Primer Sequence** | **Targeted Exon** |
| --- | --- | --- |
| *TCF4* | F: 5’-ATGGCAAATAGAGGAAGCGG  R: 5’-TGGAGAATAGATCGAAGCAAG | 13 |
| *GAPDH* | F: 5’-TGCACCACCAACTGCTTAGC  R: 5’-GGCATGGACTGTGGTCATGAG | 7 |
| *18S* | F: 5’-CCATCCAATCGGTAGTAGCG  R: 5’-GTAACCCGTTGAACCCCATT | NA |

**Table S1B**

| **Gene Name** | **TaqMan Probe ID** | **Targeted Exon** |
| --- | --- | --- |
| *IGF2* | Hs01005964_g1 | 4-5 |
| *CDKN1C* | Hs00908986_g1 | 3-4 |
| *NEUROG2* | Hs00935087_g1 | 1-2 |
| *CDK6* | Hs01026373_m1 | 5-6 |
| *BMP7* | Hs01002399_m1 | 6-7 |
| *CASP1* | Hs00354836_m1 | 7-8 |
| *CASP8* | Hs01018151_m1 | 4-5 |
| *FAS* | Hs00236330_m1 | 2-3 |
| *NTRK1* | Hs01021006_g1 | 10-11 |
| *NOTCH1* | Hs01062014_m1 | 33-34 |
| *18S* | Hs03003631_g1 | NA |

**Table S1C**

| **Gene** | **Primer sequences** |
| --- | --- |
| *BHLHE40* | F: 5’-TACCTGATCCCACCTTCAGC  R: 5’-TTGAGGCCTGGGTATAGCAC |
| *MEF2C* | F: 5’-tcgagataccCACAACACAC  R: 5’-TCGTACGAACTGCTACAGCT |
| *TCF3/E47* | F: 5’-AAGCCACTGCACACAGACAG  R: 5’-CGCATCACTTTCCACATGAC |
| *SNAI1* | F: 5’-ACAGCTGCTTTGAGCTACAG  R: 5’-gcatagttagtcacacctcg |
| *SNAI2* | F: 5’-CAGTGCAAAAACTGCTCCAA  R: 5’-GCTTCGGAGTGAAGAAATGC |
| *ZEB1* | F: 5’-GTGCACAAGAAGAGCCACAA  R: 5’-TTGCGCAAGACAAGTTCAAG |
| *ZEB2* | F: 5’-TACGGATCCCGAAACGATAC  R: 5’-CCTCGTGGTCTGATTTGGTT |
